# A phase IV study to evaluate the safety of fruquintinib in Chinese patients in real-world clinical practice

**DOI:** 10.1093/oncolo/oyae073

**Published:** 2024-04-20

**Authors:** Jin Li, Zhiqiang Wang, Haijun Zhong, Yifu He, Chen Zhang, Zuoxing Niu, Shujun Yang, Tao Zhang, Liangjun Zhu, Yongqian Shu, Yong Gao, Jianjun Peng, Yan Song, Jian Li, Ying Yuan, Haibo Zhang, Gengsheng Yu, Yunqi Hua, Jianjun Xiao, Jianfei Fu, Yulong Zheng, Hua Xue, Xian Luo, Ming Shi, Weiguo Su, Shukui Qin

**Affiliations:** Department of Medical Oncology, Tongji University Shanghai East Hospital, Shanghai, People’s Republic of China; State Key Laboratory of Oncology in South China, Collaborative Innovation Center for Cancer Medicine, Sun Yat-sen University Cancer Center, Guangzhou, People’s Republic of China; Department of Medical Oncology, Sun Yat-sen University Cancer Center, Guangzhou, People’s Republic of China; Department of Medical Oncology, Zhejiang Cancer Hospital, Hangzhou, People’s Republic of China; Department of Medical Oncology, Anhui Provincial Cancer Hospital, Hefei, People’s Republic of China; Department of Radiotherapy and Chemotherapy, Ningbo No.2 Hospital, Ningbo, People’s Republic of China; Department of Medical Oncology, Shandong Cancer Hospital, Shandong Academy of Medical Sciences, Jinan, People’s Republic of China; Department of Internal Medicine, The Affiliated Cancer Hospital of Zhengzhou University and Henan Cancer Hospital, Zhengzhou, People’s Republic of China; Cancer Center, Union Hospital, Tongji Medical College, Huazhong University of Science and Technology, Wuhan, People’s Republic of China; Jiangsu Cancer Hospital, Nanjing, People’s Republic of China; Oncology, Jiangsu Province Hospital and Nanjing Medical University First Affiliated Hospital, Nanjing, People’s Republic of China; Department of Oncology, Shanghai East Hospital, Tongji University School of Medicine, Shanghai, People’s Republic of China; Center of Gastrointestinal Surgery, The First Affiliated Hospital, Sun Yat-sen University, Guangzhou, People’s Republic of China; Department of Medical Oncology, National Cancer Center/National Clinical Research Center for Cancer/Cancer Hospital, Chinese Academy of Medical Sciences and Peking Union Medical College, Beijing, People’s Republic of China; Department of Gastrointestinal Oncology, Peking University Cancer Hospital and Institute, Beijing, People’s Republic of China; Department of Medical Oncology, The Second Affiliated Hospital, Zhejiang University School of Medicine, Hangzhou, People’s Republic of China; Guangdong Provincial Hospital of Chinese Medicine, The Second Affiliated Hospital of Guangzhou University of Chinese Medicine, Guangdong Provincial Academy of Chinese Medical Sciences, Guangzhou, People’s Republic of China; Department of Medical Oncology, Jiangmen Central Hospital, Jiangmen, People’s Republic of China; Department of Medical Oncology, Baotou Tumor Hospital, Baotou, People’s Republic of China; Department of Oncology, Zhongshan City People’s Hospital, Zhongshan, People’s Republic of China; Department of Medical Oncology, Jinhua Central Hospital, Jinhua, People’s Republic of China; Department of Medical Oncology, The First Affiliated Hospital, Zhejiang University School of Medicine, Hangzhou, People’s Republic of China; HUTCHMED Limited, Shanghai, People’s Republic of China; HUTCHMED Limited, Shanghai, People’s Republic of China; HUTCHMED Limited, Shanghai, People’s Republic of China; HUTCHMED Limited, Shanghai, People’s Republic of China; Gastrointestinal Cancer Center of Nanjing Tianyinshan Hospital, China Pharmaceutical University, Jiangsu, People’s Republic of China

**Keywords:** fruquintinib, colorectal cancer, safety, VEGF, Chinese

## Abstract

**Introduction:**

Fruquintinib is approved in China for patients with metastatic colorectal cancer (CRC) who progressed after 2 lines of chemotherapy. This postmarketing study was conducted to evaluate the safety of fruquintinib in the Chinese population, including previously treated patients with advanced CRC and other solid tumors.

**Methods:**

Patients in the first cycle of fruquintinib or expected to start fruquintinib within a week were enrolled. Fruquintinib was administrated according to the label or per physicians’ discretion. Patient characteristics and safety information were collected at baseline, 1 month, and 6 months after consent (or 30 days after the last dose).

**Results:**

Overall, 3005 patients enrolled between April 24, 2019 and September 27, 2022. All enrolled patients received at least one dose of fruquintinib. Most patients had metastases at baseline. The median age was 60 years. More than half (64.0%) of the patients started fruquintinib at 5 mg, and the median treatment exposure was 2.7 months. Nearly one-third (32.5%) of patients with CRC received fruquintinib with concomitant antineoplastic agents. Treatment-emergent adverse events (TEAEs) leading to dose modification were reported in 626 (20.8%) patients, and 469 (15.6%) patients experienced TEAEs leading to treatment discontinuation. The most common grade ≥ 3 TEAEs were hypertension (6.6%), palmar-plantar erythrodysesthesia syndrome (2.2%), and platelet count decreased (1.0%). Combination therapy did not lead to excessive toxicities.

**Conclusions:**

The safety profile of fruquintinib in the real world was generally consistent with that in clinical studies, and the incidence of TEAEs was numerically lower than known VEGF/VEGFR inhibitor-related AEs. Fruquintinib exhibited manageable safety and tolerability in Chinese patients in the real-world setting.

**Implications for Practice**

While fruquintinib is approved in China for metastatic colorectal cancer that progressed after ≥2 lines of standard systemic therapy, safety information from clinical studies may not comprehensively reflect clinical practice in the real world. This study with over 3000 patients showed that the safety profile of fruquintinib in the real world was generally consistent with that from clinical studies, and the incidence of adverse events (AEs) was numerically lower than the known VEGF/VEGFR inhibitor-related AEs.

## Introduction

Colorectal cancer (CRC) is the third most common cancer, with almost 2 million newly diagnosed cases and the second most common cause of cancer death in 2020 globally.^1^ China had the largest number of new diagnoses and deaths due to CRC, which accounted for 28.2% (521 490) of new cases and 28.1% (247 563) of deaths worldwide in 2018.^2^ Despite improvements in effective screening techniques, approximately 20% of CRC cases are diagnosed at the metastatic stage.^3^ Furthermore, >50% of patients with CRC develop metastases,^4^ the most common sites of metastasis being the liver and lungs.^5,6^ Patients with metastatic CRC are treated with chemotherapy alone or in combination with targeted therapy or immunotherapy based on molecular subtypes of the disease (eg, microsatellite instability-high/mismatch repair-deficient CRC).^7,8^

The development of targeted therapy focuses on the inhibition of tumorigenesis and related signaling pathways in the tumor microenvironment.^9^ Angiogenesis is critical in the development and progression of malignant tumors and vascular endothelial growth factor (VEGF)/VEGF receptor (VEGFR) signaling is one of the most predominant pathways for tumor angiogenesis.^9^ In the past 2 decades, therapies that target VEGF/VEGFR have been approved by the US Food and Drug Administration for patients with metastatic CRC. Fruquintinib is a potent and highly selective small-molecule tyrosine kinase inhibitor of VEGFR-1, VEGFR-2, and VEGFR-3 approved in China for patients with metastatic CRC who progressed after 2 lines of chemotherapy.^10^

Currently, the safety profile of fruquintinib is derived mainly from clinical trials^11-13^ or retrospective studies.^14^ However, data from clinical studies with the selected study populations may not comprehensively reflect the clinical experience in the real world.^15^ This is a postmarketing study conducted to evaluate the safety of fruquintinib in the Chinese population, including previously treated patients with advanced CRC and other solid tumors.

## Materials and methods

### Study design and patients

This was a prospective, multicenter, postmarketing study to evaluate the safety of fruquintinib in Chinese patients. The study was conducted across 96 clinical sites in patients who were either in the first cycle of fruquintinib or expected to start fruquintinib within 1 week. Patients who were eligible for fruquintinib treatment per physicians’ discretion were enrolled. Three scheduled study visits at baseline, 1 month, and 6 months after consent (or 30 days after the last dose, whichever occurred first) were planned to collect patient characteristics and safety information.

### Ethics approval and consent to participate

The study was conducted in accordance with the Declaration of Helsinki and Good Clinical Practice for Drugs in China. The study protocol and its amendments were reviewed and approved by the ethics committees. All patients provided informed consent before study participation.

### Treatment

Fruquintinib was administered orally according to the approved label (5 mg once daily at the same time each day continuously for 21 days in a 28-day dosing cycle [3-week treatment followed by a 1-week drug-free period]) or per physicians’ discretion. Dose interruption should be considered first for patients who required dose modification due to adverse events (AEs). Treatment could resume after drug interruption at the original dose if the AE was resolved to grade ≤ 1 within 1 week or at a lower dose (1 mg reduction) if it was resolved within 2 weeks. Fruquintinib was discontinued permanently if the patient experienced intolerance with fruquintinib 3 mg daily. Principles of dose adjustment for fruquintinib are in the Supplementary Material (p 2).

### Endpoints and assessments

Study endpoints were incidence of AEs and serious AEs and severity of AEs graded per National Cancer Institute Common Terminology Criteria for Adverse Events version 4.03.

### Statistical analysis

The study planned to collect safety information from ≥3000 patients who received ≥1 dose of fruquintinib after enrollment, based on the technical requirements specified in the Guidelines for Drug Manufacturers on Intensive Drug Monitoring (2013, No 12) in China^16^ and the preliminary safety profile of fruquintinib.

All enrolled patients who received ≥1 dose of fruquintinib were included in the safety analysis set (SS).

Statistical analyses were done in the SS, mainly descriptive, and summaries were presented by the CRC group, non-CRC group, and overall population. Subgroup analyses were performed for age (<65 vs ≥65 years), tumor metastases (lung vs liver vs lung and liver), and initial dose of fruquintinib (5 mg vs 4 mg vs 3 mg). Post hoc analyses were done in subgroups of treatment combination (fruquintinib monotherapy vs combination therapy) in the CRC group and baseline Eastern Cooperative Oncology Group (ECOG) performance status score (0-1 vs ≥2). Statistical analyses were performed using SAS version 9.4 (or above). This study is registered at ClinicalTrials.gov, NCT04005066.

## Results

### Patients

**Figure 1.**
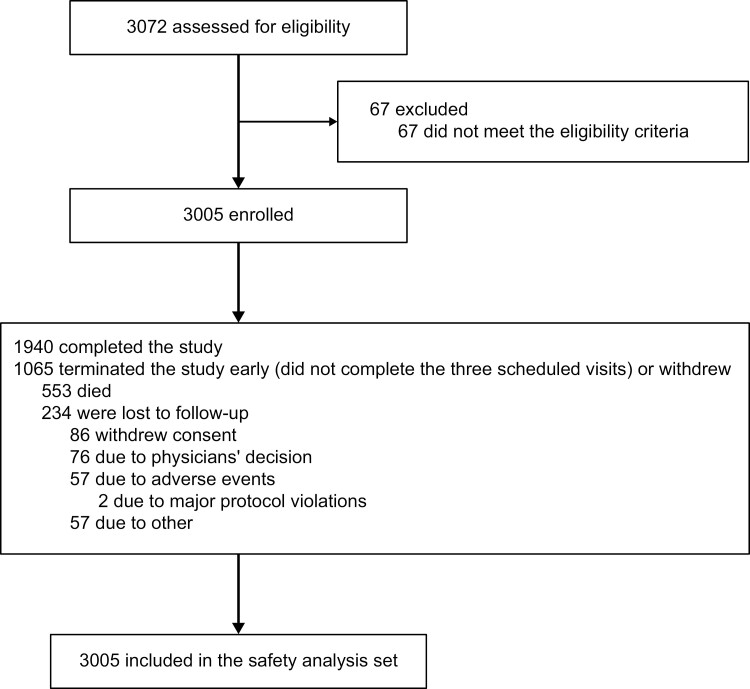
Trial profile.

Between April 24, 2019 and September 27, 2022, 3005 patients were enrolled and included in the SS (Figure 1). Three patients had unknown primary tumors (patient 1: unknown origin, likely gastrointestinal tract, with lung metastasis; patient 2: abdominal metastasis of unknown primary tumor; patient 3: multicentric intestinal adenocarcinoma with peritoneal, lymph node, and lung metastasis). All other patients (*n* = 3002) were in either the CRC group (*n* = 2798) or non-CRC group (*n* = 204) based on their primary tumor. Primary diagnoses of the non-CRC group are summarized in Supplementary Table S1.

All patients were Chinese, and the median age was 60.0 years, with 1053 (35%) patients ≥65 years old (Table 1). Among 2362 patients whose tumor metastases’ locations were collected, 1733 (73.4%) had liver metastases, 1238 (52.4%) had lung metastases, and 609 (25.8%) had both liver and lung metastases.

**Table 1.** Demographic and baseline characteristics.

	Colorectal cancer (*n* = 2798)	Non-colorectal cancer (*n* = 204)	Total (*N* = 3005^a^)
Age, median (IQR), years	60.0 (52.0-67.0)	58.0 (52.5-67.0)	60.0 (52.0-67.0)
Age group, *n* (%)			
<65 years	1811 (64.7)	139 (68.1)	1952 (65.0)
≥65 years	987 (35.3)	65 (31.9)	1053 (35.0)
Male, *n* (%)	1658 (59.3)	135 (66.2)	1795 (59.7)
Chinese, *n* (%)	2798 (100.0)	204 (100.0)	3005 (100.0)
BMI			
*N* (missing)	2751 (47)	202 (2)	2956 (49)
Median (IQR), kg/m^2^	22.6 (20.3-24.9)	21.1 (19.0-23.8)	22.6 (20.2-24.8)
Pathologic type, *n* (%)			
Adenocarcinoma	2703 (96.6)	162 (79.4)	2868 (95.4)
Mucinous carcinoma	19 (0.7)	2 (1.0)	21 (0.7)
Undifferentiated carcinoma	0	1 (0.5)	1 (0.0)
Other	76 (2.7)	39 (19.1)	115 (3.8)
Tumor metastasis			
*N* (missing)	2259 (539)	101 (103)	2362 (643)
Liver, *n* (%)	1660 (73.5)	73 (72.3)	1733 (73.4)
Lung, *n* (%)	1200 (53.1)	36 (35.6)	1238 (52.4)
Liver and lung, *n* (%)	601 (26.6)	8 (7.9)	609 (25.8)
Time from initial diagnosis to study enrollment			
*N* (missing)	2783 (15)	204 (0)	2990 (15)
Median (IQR), months	21.5 (13.1-36.4)	7.5 (0.1-21.5)	20.8 (12.2-35.7)
Time from initial diagnosis to first dose of fruquintinib			
*N* (missing)	2783 (15)	204 (0)	2990 (15)
Median (IQR), months	21.4 (13.1-36.2)	7.5 (0.1-21.2)	20.7 (12.1-35.7)

^a^Included 3 patients who had unknown primary tumors: patient 1 had an unknown origin, likely gastrointestinal tract, with lung metastasis; patient 2 had abdominal metastasis of unknown primary tumor; patient 3 had multicentric intestinal adenocarcinoma with peritoneal, lymph node, and lung metastasis.

Abbreviations: BMI, body mass index; IQR, interquartile range.

### Exposure to fruquintinib

In the SS, 1923 (64.0%) patients started treatment with the initial dose of fruquintinib at 5 mg; median treatment exposure was 2.7 months (interquartile range: 1.3-5.7 months; Table 2). The median relative dose intensity was 85.3% among 2833 patients with evaluable data.

**Table 2.** Treatment exposure and dose modification.

	Colorectal cancer (*n* = 2798)	Non-colorectal cancer (*n* = 204)	Total (*N* = 3005^a^)
Initial dose level of fruquintinib (mg)			
5	1850 (66.1)	70 (34.3)	1923 (64.0)
4	288 (10.3)	15 (7.4)	303 (10.1)
3	620 (22.2)	110 (53.9)	730 (24.3)
≤2	40 (1.4)	9 (4.4)	49 (1.6)
Total exposure, median (IQR), months	2.7 (1.4-5.7)	1.7 (1.3-4.3)	2.7 (1.3-5.7)
<6 months, *n* (%)	2207 (78.9)	172 (84.3)	2382 (79.3)
≥6 months, *n* (%)	591 (21.1)	32 (15.7)	623 (20.7)
Relative dose intensity^b^^,^^c^			
*N*	2633	197	2833
Median (IQR), %	85.3 (61.3-101.3)	56.0 (53.3-90.7)	85.3 (61.3-101.3)
At least 1 dose modification, *n* (%)	923 (33.0)	51 (25.0)	975 (32.4)
Dose reduced	439 (15.7)	21 (10.3)	460 (15.3)
Withhold medication	522 (18.7)	31 (15.2)	554 (18.4)
Dose increased	166 (5.9)	7 (3.4)	173 (5.8)
Reason for dose modification, *n* (%)			
AEs	619 (22.1)	20 (9.8)	639 (21.3)
Other	434 (15.5)	34 (16.7)	469 (15.6)

^a^Included 3 patients who had unknown primary tumors: patient 1 had an unknown origin, likely gastrointestinal tract, with lung metastasis; patient 2 had abdominal metastasis of unknown primary tumor; patient 3 had multicentric intestinal adenocarcinoma with peritoneal, lymph node, and lung metastasis.

^b^Cumulative dose, dose intensity, and relative dose intensity could not be calculated due to missing start and end dates of multiple consecutive medication records in some patients.

^c^Relative dose intensity = dose intensity (mg/day)/planned dose intensity (mg/day). When the recommended regimen in the approved label is followed, the dose intensity is (5 mg × 21)/28 = 3.75 mg/day.

Abbreviations: AE, adverse event; IQR, interquartile range.

The initial dose of fruquintinib by subgroups is summarized in Supplementary Table S2. Patients aged <65 years were more likely to start fruquintinib at 5 mg (67.9% vs 56.7%) compared with patients aged ≥65 years. Patients with higher baseline ECOG performance status score (≥2) were more likely to be given the personalized dose regimen compared with those with lower baseline ECOG performance status score (0-1; 30.3% vs 23.9%). Among patients with CRC, the starting dose of fruquintinib was consistent, regardless of whether it was used as a monotherapy or combination therapy. A higher percentage of patients who received combination treatment with chemotherapy started fruquintinib at 5 mg compared with those who received combination treatment with immunotherapy (68.0% vs 58.2%). A lower percentage of non-CRC patients started fruquintinib at 5 mg versus CRC patients (34.3% vs 66.1%). The site of tumor metastasis did not influence the initial dose of fruquintinib.

Treatment duration was generally consistent across subgroups by age and initial dose (Supplementary Table S3). Longer treatment duration was observed in fruquintinib combination therapy versus monotherapy (median: 3.5 vs 2.5 months) and patients with lung metastases (medians of lung vs liver vs lung and liver: 3.0 vs 2.5 vs 2.6 months). Relative dose intensity of fruquintinib was similar across all subgroups except by age (≥65 vs <65 years: 80.0% vs 88.0%) and primary tumor type (CRC vs non-CRC: 85.3% vs 56.0%).

### Safety

In the SS, 2291 (76.2%) patients experienced treatment-emergent AEs (TEAEs) and 718 (23.9%) patients had grade ≥ 3 AEs (Table 3). TEAEs leading to dose modification were reported in 626 (20.8%) patients (interruption: 320 [10.7%], reduction: 348 [11.6%] patients), and 469 (15.6%) patients experienced TEAEs leading to treatment discontinuation. TEAEs leading to death were reported in 169 (5.6%) patients. TEAEs related to fruquintinib were reported in 1901 (63.3%) patients: 418 (13.9%) patients experienced grade ≥ 3 treatment-related AEs (TRAEs), 562 (18.7%) required dose modification (interruption: 264 [8.8%], reduction: 331 [11.0%] patients) and 362 (12.1%) required treatment discontinuation. TRAEs leading to death were reported in 8 (0.3%) patients.

**Table 3.** Summary of adverse events.

*n* (%)	Colorectal cancer (*n* = 2798)	Non-colorectal cancer (*n* = 204)	Total (*N* = 3005^a^)
Any TEAEs	2169 (77.5)	120 (58.8)	2291 (76.2)
Grade ≥ 3	690 (24.7)	28 (13.7)	718 (23.9)
Serious AE	317 (11.3)	13 (6.4)	330 (11.0)
Leading to death	162 (5.8)	7 (3.4)	169 (5.6)
Leading to dose modification	608 (21.7)	18 (8.8)	626 (20.8)
Leading to dose interruption	311 (11.1)	9 (4.4)	320 (10.7)
Leading to dose reduction	337 (12.0)	11 (5.4)	348 (11.6)
Leading to discontinuation	448 (16.0)	21 (10.3)	469 (15.6)
Any TRAEs	1812 (64.8)	87 (42.7)	1901 (63.3)
Grade ≥ 3	407 (14.6)	11 (5.4)	418 (13.9)
Serious AE	84 (3.0)	2 (1.0)	86 (2.9)
Leading to death	8 (0.3)	0	8 (0.3)
Leading to dose modification	546 (19.5)	16 (7.8)	562 (18.7)
Leading to dose interruption	257 (9.2)	7 (3.4)	264 (8.8)
Leading to dose reduction	320 (11.4)	11 (5.4)	331 (11.0)
Leading to discontinuation	347 (12.4)	15 (7.4)	362 (12.1)

^a^Included 3 patients who had unknown primary tumors: patient 1 had an unknown origin, likely gastrointestinal tract, with lung metastasis; patient 2 had abdominal metastasis of unknown primary tumor; patient 3 had multicentric intestinal adenocarcinoma with peritoneal, lymph node, and lung metastasis.

Abbreviations: AE, adverse event; TEAE, treatment-emergent adverse event; TRAE, treatment-related adverse event.

The most common TEAEs (any grade with an incidence ≥5%) in the overall population were palmar-plantar erythrodysesthesia syndrome (*n* = 594 [19.8%]), hypertension (*n* = 480 [16.0%]), asthenia (*n* = 350 [11.7%]), appetite decreased (*n* = 294 [9.8%]), diarrhea (*n* = 281 [9.4%]), dysphonia (*n* = 260 [8.7%]), proteinuria (*n* = 199 [6.6%]), abdominal pain (*n* = 190 [6.3%]), and platelet count decreased (*n* = 171 [5.7%]; Table 4).

**Table 4.** Most common (incidence ≥ 2%) treatment-emergent adverse events.

	Colorectal cancer (*n* = 2798)		Non-colorectal cancer (*n* = 204)		Total (*N* = 3005^a^)	
	Any Grade	Grade ≥ 3	Any Grade	Grade ≥ 3	Any Grade	Grade ≥ 3
Palmar-plantar erythrodysesthesia syndrome	578 (20.7)	67 (2.4)	15 (7.4)	0	594 (19.8)	67 (2.2)
Hypertension	473 (16.9)	196 (7.0)	7 (3.4)	1 (0.5)	480 (16.0)	197 (6.6)
Asthenia	340 (12.2)	19 (0.7)	10 (4.9)	0	350 (11.7)	19 (0.6)
Appetite decreased	283 (10.1)	13 (0.5)	11 (5.4)	1 (0.5)	294 (9.8)	14 (0.5)
Diarrhea	264 (9.4)	27 (1.0)	17 (8.3)	2 (1.0)	281 (9.4)	29 (1.0)
Dysphonia	252 (9.0)	1 (0.04)	7 (3.4)	0	260 (8.7)	1 (0.03)
Proteinuria	197 (7.0)	24 (0.9)	2 (1.0)	0	199 (6.6)	24 (0.8)
Abdominal pain	178 (6.4)	21 (0.8)	12 (5.9)	2 (1.0)	190 (6.3)	23 (0.8)
Vomiting	104 (3.7)	8 (0.3)	11 (5.4)	0	115 (3.8)	8 (0.3)
Constipation	106 (3.8)	0	4 (2.0)	0	110 (3.7)	0
Oral ulcer	96 (3.4)	1 (0.04)	9 (4.4)	0	105 (3.5)	1 (0.03)
Rash	85 (3.0)	5 (0.2)	4 (2.0)	1 (0.5)	89 (3.0)	6 (0.2)
Bloating	86 (3.1)	3 (0.1)	1 (0.5)	0	87 (2.9)	3 (0.1)
Nausea	80 (2.9)	3 (0.1)	5 (2.5)	0	85 (2.8)	3 (0.1)
Fever	74 (2.6)	4 (0.1)	6 (2.9)	0	80 (2.7)	4 (0.1)
Oral mucositis	60 (2.1)	8 (0.3)	6 (2.9)	1 (0.5)	66 (2.2)	9 (0.3)
Platelet count decreased	158 (5.7)	28 (1.0)	13 (6.4)	2 (1.0)	171 (5.7)	30 (1.0)
White blood cell count decreased	79 (2.8)	5 (0.2)	14 (6.9)	1 (0.5)	93 (3.1)	6 (0.2)
Cough	80 (2.9)	2 (0.1)	1 (0.5)	0	81 (2.7)	2 (0.1)
Back pain	75 (2.7)	4 (0.1)	5 (2.5)	1 (0.5)	80 (2.7)	5 (0.2)
Hypokalemia	66 (2.4)	16 (0.6)	6 (2.9)	1 (0.5)	72 (2.4)	17 (0.5)
Hypoalbuminemia	69 (2.5)	3 (0.1)	3 (1.5)	0	72 (2.4)	3 (0.1)
Arthralgia	63 (2.3)	5 (0.2)	3 (1.5)	0	66 (2.2)	5 (0.2)
Anemia	79 (2.8)	23 (0.8)	7 (3.4)	2 (1.0)	86 (2.9)	25 (0.8)
Abnormal liver function	56 (2.0)	7 (0.3)	2 (1.0)	0	58 (1.9)	7 (0.2)
Pain in extremity	54 (1.9)	7 (0.3)	2 (1.0)	0	56 (1.9)	7 (0.2)
Headache	51 (1.8)	2 (0.1)	0	0	51 (1.7)	2 (0.1)
Aspartate aminotransferase increased	44 (1.6)	4 (0.1)	5 (2.5)	0	49 (1.6)	4 (0.1)
Edema peripheral	46 (1.6)	3 (0.1)	3 (1.5)	0	49 (1.6)	3 (0.1)
Pain	44 (1.6)	1 (0.04)	3 (1.5)	0	47 (1.6)	1 (0.03)
Neutrophil count decreased	35 (1.3)	4 (0.1)	5 (2.5)	1 (0.5)	40 (1.3)	5 (0.2)
Hyponatremia	39 (1.4)	14 (0.5)	0	0	39 (1.3)	14 (0.5)

^a^Included 3 patients who had unknown primary tumors: patient 1 had an unknown origin, likely gastrointestinal tract, with lung metastasis; patient 2 had abdominal metastasis of unknown primary tumor; patient 3 had multicentric intestinal adenocarcinoma with peritoneal, lymph node, and lung metastasis.

TRAEs are summarized in Supplementary Table S4. In the SS, the common (≥1%) TRAEs leading to treatment discontinuation were palmar-plantar erythrodysesthesia syndrome (*n* = 76 [2.5%]), hypertension (*n* = 56 [1.9%]), asthenia (*n* = 35 [1.2%]), and decreased appetite (*n* = 31 [1.0%]). Common (≥1%) TRAEs leading to treatment interruption were palmar-plantar erythrodysesthesia syndrome and hypertension (*n* = 50 [1.7%] each). TRAEs leading to dose reduction that occurred in ≥1% of patients were palmar-plantar erythrodysesthesia syndrome (*n* = 114 [3.8%]), hypertension (*n* = 67 [2.2%]), and asthenia (*n* = 40 [1.3%]). Serious TRAEs that occurred in ≥0.1% of patients were hypertension (*n* = 10 [0.3%]); platelet count decreased (*n* = 7 [0.2%]); palmar-plantar erythrodysesthesia syndrome, proteinuria, upper gastrointestinal hemorrhage, abdominal pain, and decreased appetite (*n* = 4 [0.1%] each); and anemia, liver dysfunction, lower gastrointestinal hemorrhage, intestinal perforation, gastrointestinal hemorrhage, and death (*n* = 3 [0.1%]). Of note, 2 cases of nephrotic syndrome were considered serious TRAEs; however, both patients had confounding factors (hypertension, history of diabetes and abnormal baseline urinalysis, etc.). All TRAEs leading to deaths occurred in patients with CRC.

Summary of AEs by subgroup is presented in Supplementary Table S5. The incidence of AEs was generally similar across subgroups by age, primary tumor type, monotherapy or combination therapy with fruquintinib in patients with CRC, and site of tumor metastasis. Supplementary Table S6 shows TEAEs and TRAEs of monotherapy or combination therapy with fruquintinib in patients with CRC and Supplementary Table S7 presents TEAEs and TRAEs of fruquintinib in combination with immunotherapy or chemotherapy in patients with CRC. The incidence of grade ≥ 3 and serious TEAEs and TRAEs was similar across subgroups by initial treatment dose of fruquintinib.

The incidence of TEAEs and TRAEs leading to dose modification and treatment discontinuation by subgroups was generally consistent with the overall population (Supplementary Table S8). A lower incidence of TEAEs and TRAEs leading to dose modification and treatment discontinuation was observed in the non-CRC group versus the CRC group.

## Discussion

The safety profile based on randomized controlled trials may not be representative of the whole disease population due to its tightly controlled conditions.^17^ Therefore, real-world evidence is necessary to understand how well the results from randomized trials could be translated into routine clinical practice.^18^ More than half of the 96 participating centers of this study were tertiary hospitals in China, which represented China’s national and regional medical centers for cancer care.^19^

Patients enrolled in this study were representative of the clinical experience of fruquintinib in real-world clinical practice in China and provided safety information in various patient populations, including non-CRC patients. In the current study, the percentage of patients with CRC aged ≥65 years was almost double that seen in the FRESCO trial^11^ (35.3% vs 18.0%), which is more representative of the population in which the CRC incidence is higher in older patients in China.^20^ Additionally, the FRESCO trial^11^ only included patients with a baseline ECOG performance status score of 0 or 1, while the current study enrolled patients regardless of ECOG performance status score.

Most patients treated with fruquintinib experienced at least one TEAE (98.6%) and TRAE (95.7%) in the FRESCO trial,^11^ whereas a lower incidence of TEAEs (76.2%) and TRAEs (63.3%) was reported in the current study. These results could be related to the nature of a real-world study, where TEAEs were collected through voluntary reporting by patients who may not have been able to capture all AEs that occurred during the study period. The most common AEs were generally consistent with the known safety profile of fruquintinib and have been observed in other VEGF or VEGFR inhibitor therapies,^21-23^ related to the mechanisms of action.^24,25^

Overall, the common AEs during fruquintinib treatment were clinically manageable and did not affect the drug tolerability for the study population. Higher clinical tolerability for fruquintinib was observed in our current study as evidenced by fewer TEAEs leading to dose modification (20.8% vs 47.1%) compared with the incidence in the FRESCO study.^11^ Eight deaths (0.3%) were related to treatment. However, no association was identified between treatment-related deaths and a specific AE, and the incidence of treatment-related deaths was comparable with other CRC studies with VEGFR inhibitors (0.45% in the CONSIGN study).^26^

Although the approved standard dose of fruquintinib is 5 mg daily for 21 days in a 28-day dosing cycle, individualized starting doses were used in patients based on their disease and physical conditions in the current study. The starting dose of fruquintinib ranged from 3 to 5 mg, with a similar duration of treatment across all dosing groups. Adjustment of initial dose was also observed in studies of regorafenib and other anti-VEGFR tyrosine kinase inhibitors. Therefore, the selection of initial treatment dose might depend on the patient’s condition and physicians’ preference. In general, the incidence of AEs was similar across all dosing groups ranging from 3 to 5 mg. Most of the patients (64.0%) in the SS started fruquintinib at the standard dose of 5 mg, suggesting that physicians were confident in the treatment efficacy, safety profile, and management of TEAEs.

A larger proportion of younger patients started fruquintinib at 5 mg compared with those who were older, which contributed to the higher relative dose intensity observed in the former. Nevertheless, incidence of AEs, dose modifications, and treatment discontinuations were consistent between age groups, highlighting the safety of fruquintinib, even in older patients.

Among patients with CRC, those who received monotherapy or combination therapy with fruquintinib did not influence the selection of initial dose or incidence of AEs, dose modifications, and treatment discontinuations. However, longer duration of treatment was observed in the combination group, which might be related to better clinical efficacy introduced by combination therapy. In patients who were treated with combination therapy, a higher percentage of patients received fruquintinib 5 mg in combination with chemotherapy compared with those on immune checkpoint inhibitors.

Non-CRC patients had a shorter treatment duration with fruquintinib than patients with CRC, and more than half of them started at a customized dose (≤5 mg). This could be due to a lack of standard treatment options for these non-CRC patients with late-stage disease and experimental therapy was their best option at the discretion of physicians. However, our results should be interpreted with caution given that fruquintinib is not approved for other tumor types currently. Nonetheless, our study reflects the practicing patterns of physicians in the real world, where novel treatments may sometimes be used outside their intended indication for heavily treated patients with few treatment options.^27^

Site of tumor metastasis did not influence physicians’ choice for initial dose of fruquintinib and the incidence of AEs was similar among those groups. However, patients with lung metastasis may respond better to fruquintinib as they remained on treatment longer compared with those who had liver or lung and liver metastases; the results were similar to those observed in the FRESCO study.^11^

## Conclusion

In conclusion, the current study showed that the spectrum of AEs in the real world was generally consistent with that found in clinical studies and similar to the known safety profiles of VEGF/VEGFR inhibitors. Fruquintinib has a manageable safety and tolerability profile in Chinese patients, regardless of age, tumor type, monotherapy or combination therapy, site of tumor metastasis, and baseline ECOG performance status in the real-world setting.

## Supplementary Material

oyae073_suppl_Supplementary_Material

## Data Availability

Individual data will not be made available. The study protocol is available upon request from the corresponding author.
