# Supplementary material for: A phase IV study to evaluate the safety of fruquintinib in Chinese patients in real-world clinical practice
Source: Oncologist. 2024 Apr 20;29(8):e1012–9. doi: 10.1093/oncolo/oyae073 (PMC11299944; doi:10.1093/oncolo/oyae073)
Supplement: oyae073_suppl_Supplementary_Material [file oyae073_suppl_supplementary_material.docx]

***Supplementary Material***

1. **Dose adjustment principles of fruquintinib in the treatment of metastatic colorectal cancer**

| **Dose adjustment regimen** | **Occurrence of applicable adverse reactions** |
| --- | --- |
| Dose interruption | - Grade 2 haemorrhage - Grade 2 hand-foot skin reaction and recurrent stomatitis - Grade 2 platelet count decreased (50–75 × 10^9^/L) - Urinary protein ≥2.0 g/24 hours - All Grade 3 or Grade 4 adverse reactions (except for adverse reactions that require permanent discontinuation) |
| Dose reduction | - Adverse reaction(s) that resolve to grade ≤1 within 2 weeks of drug interruption |
| Dose  discontinuation | - Grade 3 or above haemorrhage - Gastrointestinal perforation, open wounds that required clinical treatment, fistula, nephrotic syndrome or hypertensive crisis - Grade 4 hepatic function abnormal or impairment (transaminase > 0 times the ULN) - Intolerance to a daily dose of 3 mg - The adverse reaction(s) is still grade >1 after more than 2 weeks of drug interruption |

Severity of adverse reactions are in accordance with U.S. National Cancer Institute (NCI) Common Terminology Criteria for Adverse Events (CTCAE) Version 4.03.

1. **Supplementary Tables**

## Supplementary Table 1. Additional demographic and baseline characteristics

|  | **Colorectal cancer**  **(n = 2798)** | **Non-colorectal cancer**  **(n = 204)** | **Total**^a^  **(N = 3005)** |
| --- | --- | --- | --- |
|  |  |  |  |
| **Clinical TNM stage** |  |  |  |
| N (missing) | 1594 (1204) | 85 (119) | 1680 (1325) |
| M, n (%) | 1594 (100) | 85 (100) | 1680 (100) |
| 0, n (%) | 26 (1.6) | 2 (2.4) | 28 (1.7) |
| 1, n (%) | 1563 (98.1) | 82 (96.5) | 1646 (98.0) |
| X, n (%) | 5 (0.3) | 1 (1.2) | 6 (0.4) |
| **Pathological TNM stage** |  |  |  |
| N (missing) | 932 (1866) | 113 (91) | 1045 (1960) |
| M, n (%) | 932 (100) | 113 (100) | 1045 (100) |
| 0, n (%) | 36 (3.9) | 9 (8.0) | 45 (4.3) |
| 1, n (%) | 889 (95.4) | 103 (91.2) | 992 (94.9) |
| X, n (%) | 7 (0.8) | 1 (0.9) | 8 (0.8) |
| **Non-colorectal cancer, n (%)** |  |  |  |
| Gastric cancer | -- | 85 (41.7) | 85 (2.8) |
| Small bowel cancer | -- | 22 (10.8) | 22 (0.7) |
| Esophageal cancer | -- | 14 (6.9) | 14 (0.5) |
| Liver cancer | -- | 13 (6.4) | 13 (0.4) |
| Pancreatic cancer | -- | 14 (6.9) | 14 (0.5) |
| Appendiceal carcinoma | -- | 11 (5.4) | 11 (0.4) |
| Biliary system neoplasms | -- | 14 (6.9) | 14 (0.5) |
| Urinary tract neoplasm | -- | 4 (2.0) | 4 (0.1) |
| Sarcoma | -- | 9 (4.4) | 9 (0.3) |
| Ovarian cancer | -- | 7 (3.4) | 7 (0.2) |
| Neuroendocrine tumor | -- | 3 (1.5) | 3 (0.1) |
| Lung cancer | -- | 1 (0.5) | 1 (0.0) |
| Other | -- | 7 (3.4) | 7 (0.2) |
| **Prior antineoplastic agents, n (%)** |  |  |  |
| Oxaliplatin | 1337 (47.8) | 28 (13.7) | 1367 (45.5) |
| Bevacizumab | 1185 (42.4) | 15 (7.4) | 1202 (40.0) |
| Capecitabine | 1167 (41.7) | 17 (8.3) | 1186 (39.5) |
| Irinotecan | 996 (35.6) | 13 (6.4) | 1011 (33.6) |
| Fluorouracil | 906 (32.4) | 15 (7.4) | 923 (30.7) |
| Raltitrexed | 404 (14.4) | 4 (2.0) | 408 (13.6) |
| Cetuximab | 395 (14.1) | 0 | 395 (13.1) |
| Irinotecan Hydrochloride | 297 (10.6) | 2 (1.0) | 299 (10.0) |
| Tegafur/gimeracil/octeracil | 160 (5.7) | 11 (5.4) | 171 (5.7) |
| Sintilimab | 135 (4.8) | 8 (3.9) | 143 (4.8) |
| Regorafenib | 137 (4.9) | 1 (0.5) | 138 (4.6) |
| Camrelizumab | 60 (2.1) | 9 (4.4) | 70 (2.3) |

^a^Included three patients who had unknown primary tumours: patient 1 had unknown origin, likely gastrointestinal tract, with lung metastasis; patient 2 had abdominal metastasis of unknown primary tumour; patient 3 had multicentric intestinal adenocarcinoma with peritoneal, lymph node and lung metastasis.

## Supplementary Table 2. Initial dose level of fruquintinib by subgroups

|  | **N** | **5 mg** | **4 mg** | **3 mg** | **≤2 mg** |
| --- | --- | --- | --- | --- | --- |
| **Age group** |  |  |  |  |  |
| <65 years | 1952 | 1326 (67.9) | 192 (9.8) | 413 (21.2) | 21 (1.1) |
| ≥65 years | 1053 | 597 (56.7) | 111 (10.5) | 317 (30.1) | 28 (2.7) |
| **Tumour type** |  |  |  |  |  |
| CRC group | 2798 | 1850 (66.1) | 288 (10.3) | 620 (22.2) | 40 (1.4) |
| Fruq. Monotherapy | 1888 | 1265 (67.0) | 189 (10.0) | 407 (21.6) | 27 (1.4) |
| Fruq. Combination therapy^a^ | 910 | 585 (64.3) | 99 (10.9) | 213 (23.4) | 13 (1.4) |
| with ICI | 493 | 287 (58.2) | 61 (12.4) | 139 (28.2) | 6 (1.2) |
| with Chemo | 359 | 244 (68.0) | 35 (9.7) | 72 (20.1) | 8 (2.2) |
| Non-CRC group | 204 | 70 (34.3) | 15 (7.4) | 110 (53.9) | 9 (4.4) |
| **Metastasis site** |  |  |  |  |  |
| Lung | 1238 | 796 (64.3) | 140 (11.3) | 281 (22.7) | 21 (1.7) |
| Liver | 1733 | 1137 (65.6) | 160 (9.2) | 413 (23.8) | 23 (1.3) |
| Lung and liver | 609 | 400 (65.7) | 69 (11.3) | 132 (21.7) | 8 (1.3) |
| **Baseline ECOG PS score**^b^ |  |  |  |  |  |
| CRC group |  |  |  |  |  |
| 0–1 | 138 | 97 (70.3) | 8 (5.8) | 33 (23.9) | 0 |
| ≥2 | 33 | 22 (66.7) | 1 (3.0) | 10 (30.3) | 0 |

^a^The immunotherapies given in combination with fruquintinib included PD-1 inhibitors sintilimab, camrelizumab, and tislelizumab, and PD-L1 inhibitors durvalumab and atezolizumab. The chemotherapy agents that were given together with fruquintinib included capecitabine, trifluridine/tipiracil, and tegafur/gimeracil/octeracil administered orally, and oxaliplatin, raltitrexed, fluorouracil, and irinotecan administered intravenously.
^b^ECOG PS score were only collected from enrolled patients under protocol version 2.0.
Chemo, chemotherapy; CRC, colorectal cancer, ECOG PS, Eastern Cooperative Oncology Group performance score; Fruq., fruquintinib; ICI, immune checkpoint inhibitor.

## Supplementary Table 3. Exposure to fruquintinib by subgroups

|  | **N** | **Median DOT**  **months (IQR)** | **Relative dose intensity**  **% (IQR)** |
| --- | --- | --- | --- |
| **Age group** |  |  |  |
| <65 years | 1952 | 2.8 (1.4–5.7) | 88.0 (64.0–101.3) |
| ≥65 years | 1053 | 2.6 (1.3–5.6) | 80.0 (61.3–98.7) |
| **Tumour type** |  |  |  |
| CRC group | 2798 | 2.7 (1.4–5.7) | 85.3 (61.3–101.3) |
| Fruq. Monotherapy | 1888 | 2.5 (1.2–5.5) | 85.3 (64.0–101.3) |
| Fruq. Combination therapy^a^ | 910 | 3.5 (1.8–5.9) | 85.3 (61.3–101.3) |
| with ICI | 493 | 3.6 (1.8–6.0) | 80.0 (61.3–98.7) |
| with Chemo | 359 | 3.8 (1.8–6.0) | 88.0 (64.0–101.3) |
| Non-CRC group | 204 | 1.7 (1.3–4.3) | 56.0 (53.3–90.7) |
| **Metastasis site** |  |  |  |
| Lung | 1238 | 3.0 (1.4–5.8) | 82.7 (61.3–101.3) |
| Liver | 1733 | 2.5 (1.3–5.4) | 85.3 (61.3–101.3) |
| Lung and liver | 609 | 2.6 (1.3–5.4) | 85.3 (64.0–101.3) |
| **Baseline ECOG PS score**^b^ |  |  |  |
| CRC group |  |  |  |
| 0–1 | 138 | 3.7 (1.4–6.0) | 90.7 (64.0–104.0) |
| ≥2 | 33 | 4.1 (1.9–6.1) | 88.0 (62.7–102.7) |
| **Initial dose level of fruquintinib** |  |  |  |
| 5 mg | 1923 | 2.7 (1.3–5.7) | 98.7 (82.7–104.0) |
| 4 mg | 303 | 2.8 (1.5–5.7) | 80.0 (72.0–82.7) |
| 3 mg | 730 | 2.5 (1.3–5.5) | 58.7 (53.3–61.3) |

^a^The immunotherapies given in combination with fruquintinib included PD-1 inhibitors sintilimab, camrelizumab, and tislelizumab, and PD-L1 inhibitors durvalumab and atezolizumab. The chemotherapy agents that were given together with fruquintinib included capecitabine, trifluridine/tipiracil, and tegafur/gimeracil/octeracil administered orally, and oxaliplatin, raltitrexed, fluorouracil, and irinotecan administered intravenously. ^b^ECOG PS score were only collected from enrolled patients under protocol version 2.0.
Chemo, chemotherapy; CRC, colorectal cancer, ECOG PS, Eastern Cooperative Oncology Group performance score; Fruq., fruquintinib; ICI, immune checkpoint inhibitor; IQR, interquartile range.

## Supplementary Table 4. Most common (incidence ≥2%) treatment-related adverse events

| n (%) | **Colorectal cancer  (n = 2798)** | | **Non-colorectal cancer  (n = 204)** | | **Total**^a^  **(N = 3005)** | |
| --- | --- | --- | --- | --- | --- | --- |
|  | **Any grade** | **Grade ≥3** | **Any grade** | **Grade ≥3** | **Any grade** | **Grade ≥3** |
| Palmar-plantar erythrodysesthesia syndrome | 569 (20.3) | 67 (2.4) | 14 (6.9) | 0 | 584 (19.4) | 67 (2.2) |
| Hypertension | 463 (16.6) | 192 (6.9) | 7 (3.4) | 1 (0.5) | 470 (15.6) | 193 (6.4) |
| Asthenia | 292 (10.4) | 14 (0.5) | 10 (4.9) | 0 | 302 (10.1) | 14 (0.5) |
| Appetite decreased | 237 (8.5) | 11 (0.4) | 10 (4.9) | 1 (0.5) | 247 (8.2) | 12 (0.4) |
| Dysphonia | 232 (8.3) | 1 (0.04) | 7 (3.4) | 0 | 240 (8.0) | 1 (0.03) |
| Diarrhoea | 218 (7.8) | 21 (0.8) | 12 (5.9) | 0 | 230 (7.7) | 21 (0.7) |
| Proteinuria | 177 (6.3) | 21 (0.8) | 2 (1.0) | 0 | 179 (6.0) | 21 (0.7) |
| Platelet count decreased | 120 (4.3) | 17 (0.6) | 12 (5.9) | 2 (1.0) | 132 (4.4) | 19 (0.6) |
| Oral ulcer | 88 (3.2) | 1 (0.04) | 7 (3.4) | 0 | 95 (3.2) | 1 (0.03) |
| Abdominal pain | 83 (3.0) | 8 (0.3) | 5 (2.5) | 1 (0.5) | 88 (2.9) | 9 (0.3) |
| Rash | 72 (2.6) | 1 (0.04) | 3 (1.5) | 1 (0.5) | 75 (2.5) | 2 (0.1) |
| Vomiting | 65 (2.3) | 3 (0.1) | 8 (3.9) | 0 | 73 (2.4) | 3 (0.1) |
| Oral mucositis | 57 (2.0) | 8 (0.3) | 6 (2.9) | 1 (0.5) | 63 (2.1) | 9 (0.3) |
| White blood cell count decreased | 49 (1.8) | 2 (0.1) | 12 (5.9) | 1 (0.5) | 61 (2.0) | 3 (0.0) |
| Aspartate aminotransferase increased | 35 (1.3) | 2 (0.1) | 5 (2.5) | 0 | 40 (1.3) | 2 (0.1) |

^a^Included three patients who had unknown primary tumours: patient 1 had unknown origin, likely gastrointestinal tract, with lung metastasis; patient 2 had abdominal metastasis of unknown primary tumour; patient 3 had multicentric intestinal adenocarcinoma with peritoneal, lymph node and lung metastasis.

## Supplementary Table 5. Summary of adverse events by subgroups

|  | **N** | **Treatment-emergent** | | | **Treatment-related** | | |
| --- | --- | --- | --- | --- | --- | --- | --- |
|  |  | **Any grade** | **Grade ≥3** | **Serious** | **Any grade** | **Grade ≥3** | **Serious** |
| **Age group** |  |  |  |  |  |  |  |
| <65 years | 1952 | 1499 (76.8) | 442 (22.6) | 203 (10.4) | 1230 (63.0) | 243 (12.5) | 50 (2.6) |
| ≥65 years | 1053 | 792 (75.2) | 276 (26.2) | 127 (12.1) | 671 (63.7) | 175 (16.6) | 36 (3.4) |
| **Tumour type** |  |  |  |  |  |  |  |
| CRC group | 2798 | 2169 (77.5) | 690 (24.7) | 317 (11.3) | 1812 (64.8) | 407 (14.6) | 84 (3.0) |
| Fruq. Monotherapy | 1888 | 1406 (74.5) | 452 (23.9) | 227 (12.0) | 1170 (62.0) | 261 (13.8) | 57 (3.0) |
| Fruq. Combination therapy^a^ | 910 | 763 (83.9) | 238 (26.2) | 90 (9.9) | 642 (70.6) | 146 (16.0) | 27 (3.0) |
| with ICI | 493 | 408 (82.8) | 124 (25.2) | 53 (10.8) | 344 (69.8) | 71 (14.4) | 15 (3.0) |
| with Chemo | 359 | 307 (85.5) | 106 (29.5) | 31 (8.6) | 257 (71.6) | 65 (18.1) | 8 (2.2) |
| Non-CRC group | 204 | 120 (58.8) | 28 (13.7) | 13 (6.4) | 87 (42.7) | 11 (5.4) | 2 (1.0) |
| **Metastasis site** |  |  |  |  |  |  |  |
| Lung | 1238 | 983 (79.4) | 308 (24.9) | 143 (11.6) | 836 (67.5) | 187 (15.1) | 41 (3.3) |
| Liver | 1733 | 1342 (77.4) | 424 (24.5) | 192 (11.1) | 1121 (64.7) | 253 (14.6) | 52 (3.0) |
| Lung and liver | 609 | 490 (80.5) | 164 (27.0) | 79 (13.0) | 411 (67.5) | 98 (16.1) | 24 (3.9) |
| **Baseline ECOG PS score**^b^ |  |  |  |  |  |  |  |
| CRC group |  |  |  |  |  |  |  |
| 0–1 | 138 | 114 (82.6) | 40 (29.0) | 13 (9.4) | 96 (69.6) | 28 (20.3) | 4 (2.9) |
| ≥2 | 33 | 22 (66.7) | 9 (27.3) | 7 (21.2) | 16 (48.5) | 2 (6.1) | 1 (3.0) |
| **Initial dose level of fruquintinib** |  |  |  |  |  |  |  |
| 5 mg | 1923 | 1525 (79.3) | 501 (26.1) | 228 (11.9) | 1283 (66.7) | 302 (15.7) | 60 (3.1) |
| 4 mg | 303 | 239 (78.9) | 74 (24.4) | 29 (9.6) | 203 (67.0) | 46 (15.2) | 8 (2.6) |
| 3 mg | 730 | 494 (67.7) | 135 (18.5) | 67 (9.2) | 387 (53.0) | 68 (9.3) | 18 (2.5) |

^a^The immunotherapies given in combination with fruquintinib included PD-1 inhibitors sintilimab, camrelizumab, and tislelizumab, and PD-L1 inhibitors durvalumab and atezolizumab. The chemotherapy agents that were given together with fruquintinib included capecitabine, trifluridine/tipiracil, and tegafur/gimeracil/octeracil administered orally, and oxaliplatin, raltitrexed, fluorouracil, and irinotecan administered intravenously. ^b^ECOG PS score were only collected from enrolled patients under protocol version 2.0.
Chemo, chemotherapy; CRC, colorectal cancer, ECOG PS, Eastern Cooperative Oncology Group performance score; Fruq., fruquintinib; ICI, immune checkpoint inhibitor.

## Supplementary Table 6. Adverse events (incidence ≥2%) in the CRC group by monotherapy or combination therapy of fruquintinib

| n (%) | **Treatment-emergent** | | | | **Treatment-related** | | | |
| --- | --- | --- | --- | --- | --- | --- | --- | --- |
|  | **Fruq. monotherapy**  **(n = 1888)** | | **Fruq. combination therapy^a^**  **(n = 910)** | | **Fruq. monotherapy**  **(n = 1888)** | | **Fruq. combination therapy^a^**  **(n = 910)** | |
|  | **Any grade** | **Grade ≥3** | **Any grade** | **Grade ≥3** | **Any grade** | **Grade ≥3** | **Any grade** | **Grade ≥3** |
| Palmar-plantar erythrodysaesthesia syndrome | 376 (19.9) | 48 (2.5) | 202 (22.2) | 19 (2.1) | 372 (19.7) | 48 (2.5) | 197 (21.7) | 19 (2.1) |
| Hypertension | 328 (17.4) | 130 (6.9) | 145 (15.9) | 66 (7.3) | 321 (17.0) | 127 (6.7) | 142 (15.6) | 65 (7.1) |
| Asthenia | 224 (11.9) | 13 (0.7) | 116 (12.8) | 6 (0.7) | 190 (10.1) | 9 (0.5) | 102 (11.2) | 5 (0.6) |
| Decreased appetite | 183 (9.7) | 10 (0.5) | 100 (11.0) | 3 (0.3) | 150 (7.9) | 8 (0.4) | 87 (9.6) | 3 (0.3) |
| Dysphonia | 159 (8.4) | 0 | 93 (10.2) | 1 (0.1) | 148 (7.8) | 0 | 84 (9.2) | 1 (0.1) |
| Diarrhoea | 158 (8.4) | 12 (0.6) | 106 (11.7) | 15 (1.7) | 137 (7.3) | 9 (0.5) | 81 (8.9) | 12 (1.3) |
| Proteinuria | 115 (6.1) | 17 (0.9) | 82 (9.0) | 7 (0.8) | 102 (5.4) | 14 (0.7) | 75 (8.2) | 7 (0.8) |
| Abdominal pain | 110 (5.8) | 16 (0.9) | 68 (7.5) | 5 (0.6) | 57 (3.0) | 8 (0.4) | 26 (2.9) | 0 |
| Platelet count decreased | 87 (4.6) | 18 (1.0) | 71 (7.8) | 10 (1.1) | 74 (3.9) | 12 (0.6) | 46 (5.1) | 5 (0.6) |
| Mouth ulceration | 70 (3.7) | 1 (0.1) | 26 (2.9) | 0 | 66 (3.5) | 1 (0.1) | 22 (2.4) | 0 |
| Vomiting | 59 (3.1) | 5 (0.3) | 45 (5.0) | 3 (0.3) | 40 (2.1) | 2 (0.1) | 25 (2.8) | 1 (0.1) |
| Constipation | 53 (2.8) | 0 | 53 (5.8) | 0 | 19 (1.0) | 0 | 17 (1.9) | 0 |
| Nausea | 52 (2.8) | 2 (0.1) | 28 (3.1) | 1 (0.1) | 32 (1.7) | 2 (0.1) | 20 (2.2) | 1 (0.1) |
| Cough | 50 (2.7) | 1 (0.1) | 30 (3.3) | 1 (0.1) | 19 (1.0) | 1 (0.1) | 14 (1.5) | 1 (0.1) |
| Abdominal distension | 49 (2.6) | 3 (0.2) | 37 (4.1) | 0 | 23 (1.2) | 0 | 19 (2.1) | 0 |
| Rash | 48 (2.5) | 2 (0.1) | 37 (4.1) | 3 (0.3) | 42 (2.2) | 1 (0.1) | 30 (3.3) | 0 |
| Back pain | 46 (2.4) | 2 (0.1) | 29 (3.2) | 2 (0.2) | 28 (1.5) | 1 (0.1) | 19 (2.1) | 1 (0.1) |
| Stomatitis | 42 (2.2) | 4 (0.2) | 18 (2.0) | 4 (0.4) | 39 (2.1) | 4 (0.2) | 18 (2.0) | 4 (0.4) |
| Pyrexia | 41 (2.2) | 3 (0.2) | 33 (3.6) | 1 (0.1) | 11 (0.6) | 0 | 5 (0.6) | 0 |
| Arthralgia | 40 (2.1) | 4 (0.2) | 23 (2.5) | 1 (0.1) | 29 (1.5) | 3 (0.2) | 17 (1.9) | 1 (0.1) |
| Anaemia | 38 (2.0) | 11 (0.6) | 41 (4.5) | 12 (1.3) | 18 (1.0) | 5 (0.3) | 17 (1.9) | 4 (0.4) |
| Hypoalbuminaemia | 36 (1.9) | 3 (0.2) | 33 (3.6) | 0 | 14 (0.7) | 1 (0.1) | 7 (0.8) | 0 |
| Hypokalaemia | 36 (1.9) | 10 (0.5) | 30 (3.3) | 6 (0.7) | 8 (0.4) | 1 (0.1) | 4 (0.4) | 2 (0.2) |
| White blood cell count decreased | 34 (1.8) | 3 (0.2) | 45 (5.0) | 2 (0.2) | 26 (1.4) | 1 (0.1) | 23 (2.5) | 1 (0.1) |
| Hepatic function abnormal | 30 (1.6) | 4 (0.2) | 26 (2.9) | 3 (0.3) | 12 (0.6) | 0 | 19 (2.1) | 3 (0.3) |
| Aspartate aminotransferase increased | 22 (1.2) | 2 (0.1) | 22 (2.4) | 2 (0.2) | 17 (0.9) | 1 (0.1) | 18 (2.0) | 1 (0.1) |
| Alanine aminotransferase increased | 19 (1.0) | 1 (0.1) | 19 (2.1) | 3 (0.3) | 14 (0.7) | 0 | 13 (1.4) | 1 (0.1) |
| Hyponatraemia | 19 (1.0) | 6 (0.3) | 20 (2.2) | 8 (0.9) | 1 (0.1) | 0 | 6 (0.7) | 2 (0.2) |
| Hypothyroidism | 18 (1.0) | 0 | 24 (2.6) | 0 | 16 (0.9) | 0 | 5 (0.6) | 0 |
| Hypoproteinaemia | 16 (0.9) | 0 | 21 (2.3) | 2 (0.2) | 9 (0.5) | 0 | 4 (0.4) | 0 |
| Neutrophil count decreased | 12 (0.6) | 2 (0.1) | 23 (2.5) | 2 (0.2) | 9 (0.5) | 2 (0.1) | 10 (1.1) | 0 |
| Gamma-glutamyltransferase increased | 11 (0.6) | 3 (0.2) | 21 (2.3) | 5 (0.6) | 5 (0.3) | 1 (0.1) | 12 (1.3) | 3 (0.3) |

^a^The immunotherapies given in combination with fruquintinib included PD-1 inhibitors sintilimab, camrelizumab, and tislelizumab, and PD-L1 inhibitors durvalumab and atezolizumab. The chemotherapy agents that were given together with fruquintinib included capecitabine, trifluridine/tipiracil, and tegafur/gimeracil/octeracil administered orally, and oxaliplatin, raltitrexed, fluorouracil, and irinotecan administered intravenously.

Chemo, chemotherapy; CRC, colorectal cancer, Fruq., fruquintinib; ICI, immune checkpoint inhibitor.

## Supplementary Table 7. Adverse events (incidence ≥2%) in the CRC group by fruquintinib in combination with immunotherapy or chemotherapy

| n (%) | **TEAE** | | | | **TRAE** | | | |
| --- | --- | --- | --- | --- | --- | --- | --- | --- |
|  | **Fruq. with ICI^a^**  **(n = 493)** | | **Fruq. with Chemo^b^**  **(n = 359)** | | **Fruq. with ICI^a^**  **(n = 493)** | | **Fruq. with Chemo^b^**  **(n = 359)** | |
|  | **Any grade** | **Grade ≥3** | **Any grade** | **Grade ≥3** | **Any grade** | **Grade ≥3** | **Any grade** | **Grade ≥3** |
| Palmar-plantar erythrodysaesthesia syndrome | 121 (24.5) | 9 (1.8) | 76 (21.2) | 6 (1.7) | 119 (24.1) | 9 (1.8) | 74 (20.6) | 6 (1.7) |
| Hypertension | 78 (15.8) | 33 (6.7) | 64 (17.8) | 35 (9.8) | 78 (15.8) | 33 (6.7) | 61 (17.0) | 34 (9.5) |
| Diarrhoea | 55 (11.2) | 6 (1.2) | 42 (11.7) | 6 (1.7) | 40 (8.1) | 4 (0.8) | 34 (9.5) | 5 (1.4) |
| Dysphonia | 58 (11.8) | 0 | 34 (9.5) | 1 (0.3) | 52 (10.6) | 0 | 31 (8.6) | 1 (0.3) |
| Asthenia | 51 (10.3) | 3 (0.6) | 58 (16.2) | 2 (0.6) | 43 (8.7) | 2 (0.4) | 53 (14.8) | 2 (0.6) |
| Decreased appetite | 48 (9.7) | 2 (0.4) | 48 (13.4) | 0 | 40 (8.1) | 2 (0.4) | 42 (11.7) | 0 |
| Proteinuria | 40 (8.1) | 3 (0.6) | 29 (8.1) | 2 (0.6) | 35 (7.1) | 3 (0.6) | 27 (7.5) | 2 (0.6) |
| Platelet count decreased | 35 (7.1) | 2 (0.4) | 31 (8.6) | 4 (1.1) | 27 (5.5) | 1 (0.2) | 17 (4.7) | 1 (0.3) |
| Abdominal pain | 30 (6.1) | 2 (0.4) | 34 (9.5) | 2 (0.6) | 12 (2.4) | 0 | 17 (4.7) | 0 |
| Constipation | 30 (6.1) | 0 | 23 (6.4) | 0 | 11 (2.2) | 0 | 7 (2.0) | 0 |
| Rash | 24 (4.9) | 2 (0.4) | 16 (4.5) | 1 (0.3) | 19 (3.9) | 0 | 13 (3.6) | 0 |
| Cough | 21 (4.3) | 0 | 11 (3.1) | 1 (0.3) | 10 (2.0) | 0 | 5 (1.4) | 1 (0.3) |
| Anaemia | 20 (4.1) | 7 (1.4) | 23 (6.4) | 7 (2.0) | 11 (2.2) | 2 (0.4) | 9 (2.5) | 2 (0.6) |
| Abdominal distension | 19 (3.9) | 0 | 15 (4.2) | 0 | 9 (1.8) | 0 | 7 (2.0) | 0 |
| Hypoalbuminaemia | 19 (3.9) | 0 | 13 (3.6) | 0 | 4 (0.8) | 0 | 0 | 0 |
| White blood cell count decreased | 19 (3.9) | 1 (0.2) | 24 (6.7) | 1 (0.3) | 9 (1.8) | 0 | 11 (3.1) | 1 (0.3) |
| Hypokalaemia | 18 (3.8) | 5 (1.0) | 13 (3.6) | 2 (0.6) | 3 (0.6) | 2 (0.4) | 2 (0.6) | 0 |
| Hepatic function abnormal | 18 (3.7) | 3 (0.6) | 8 (2.2) | 0 | 12 (2.4) | 3 (0.6) | 6 (1.7) | 0 |
| Hypothyroidism | 18 (3.7) | 0 | 5 (1.4) | 0 | 4 (0.8) | 0 | 1 (0.3) | 0 |
| Vomiting | 18 (3.7) | 1 (0.2) | 25 (7.0) | 1 (0.3) | 10 (2.0) | 0 | 13 (3.6) | 0 |
| Back pain | 16 (3.3) | 0 | 12 (3.3) | 1 (0.3) | 12 (2.4) | 0 | 9 (2.5) | 1 (0.3) |
| Hypoproteinaemia | 16 (3.3) | 1 (0.2) | 10 (2.8) | 2 (0.6) | 4 (0.8) | 0 | 0 | 0 |
| Mouth ulceration | 16 (3.3) | 0 | 9 (2.5) | 0 | 14 (2.8) | 0 | 7 (2.0) | 0 |
| Alanine aminotransferase increased | 12 (2.4) | 2 (0.4) | 7 (2.0) | 0 | 8 (1.6) | 1 (0.2) | 4 (1.1) | 0 |
| Arthralgia | 12 (2.4) | 0 | 9 (2.5) | 1 (0.3) | 9 (1.8) | 0 | 7 (2.0) | 1 (0.3) |
| Aspartate aminotransferase increased | 12 (2.4) | 2 (0.4) | 7 (2.0) | 1 (0.3) | 10 (2.0) | 1 (0.2) | 6 (1.7) | 1 (0.3) |
| Hyponatraemia | 12 (2.4) | 6 (1.2) | 8 (2.2) | 2 (0.6) | 5 (1.0) | 2 (0.4) | 0 | 0 |
| Pain | 12 (2.4) | 0 | 6 (1.7) | 0 | 6 (1.2) | 0 | 2 (0.6) | 0 |
| Pyrexia | 12 (2.4) | 0 | 14 (3.9) | 1 (0.3) | 2 (0.4) | 0 | 3 (0.8) | 0 |
| Chest discomfort | 11 (2.2) | 0 | 5 (1.4) | 0 | 6 (1.2) | 0 | 3 (0.8) | 0 |
| Gamma-glutamyltransferase increased | 11 (2.2) | 4 (0.8) | 8 (2.2) | 2 (0.6) | 5 (1.0) | 2 (0.4) | 6 (1.7) | 2 (0.6) |
| Nausea | 11 (2.2) | 0 | 12 (3.3) | 0 | 7 (1.4) | 0 | 8 (2.2) | 0 |
| Peripheral oedema | 11 (2.2) | 3 (0.6) | 8 (2.2) | 1 (0.3) | 1 (0.2) | 0 | 4 (1.1) | 0 |
| Pruritus | 11 (2.2) | 0 | 4 (1.1) | 0 | 7 (1.4) | 0 | 3 (0.8) | 0 |
| Gingival pain | 10 (2.0) | 0 | 5 (1.4) | 0 | 8 (1.6) | 0 | 5 (1.4) | 0 |
| Headache | 10 (2.0) | 0 | 6 (1.7) | 0 | 7 (1.4) | 0 | 4 (1.1) | 0 |
| Blood bilirubin increased | 9 (1.8) | 2 (0.4) | 9 (2.5) | 1 (0.3) | 4 (0.8) | 1 (0.2) | 5 (1.4) | 0 |
| Insomnia | 9 (1.8) | 0 | 8 (2.2) | 0 | 3 (0.6) | 0 | 3 (0.8) | 0 |
| Urinary tract infection | 9 (1.8) | 1 (0.2) | 8 (2.2) | 2 (0.6) | 3 (0.6) | 1 (0.2) | 4 (1.1) | 2 (0.6) |
| Epistaxis | 8 (1.6) | 0 | 8 (2.2) | 0 | 5 (1.0) | 0 | 6 (1.7) | 0 |
| Pain in extremity | 7 (1.4) | 2 (0.4) | 8 (2.2) | 0 | 7 (1.4) | 2 (0.4) | 6 (1.7) | 0 |
| Upper abdominal pain | 7 (1.4) | 0 | 8 (2.2) | 1 (0.3) | 3 (0.6) | 0 | 4 (1.1) | 0 |
| Neutrophil count decreased | 6 (1.2) | 0 | 16 (4.5) | 2 (0.6) | 3 (0.6) | 0 | 5 (1.4) | 0 |
| Stomatitis | 6 (1.2) | 1 (0.2) | 14 (3.9) | 3 (0.8) | 6 (1.2) | 1 (0.2) | 14 (3.9) | 3 (0.8) |

^a^The immunotherapies given in combination with fruquintinib included PD-1 inhibitors sintilimab, camrelizumab, and tislelizumab, and PD-L1 inhibitors durvalumab and atezolizumab.
^b^The chemotherapy agents that were given together with fruquintinib included capecitabine, trifluridine/tipiracil, and tegafur/gimeracil/octeracil administered orally, and oxaliplatin, raltitrexed, fluorouracil, and irinotecan administered intravenously.
Chemo, chemotherapy; CRC, colorectal cancer, Fruq., fruquintinib; ICI, immune checkpoint inhibitor.

## Supplementary Table 8. Adverse events leading to dose modification and treatment discontinuation by subgroups

|  | **N** | **TEAE induced** | | | **TRAE induced** | | |
| --- | --- | --- | --- | --- | --- | --- | --- |
|  |  | **Dose**  **interruption** | **Dose**  **reduction** | **Dose**  **discontinuation** | **Dose**  **interruption** | **Dose**  **reduction** | **Dose**  **discontinuation** |
| **Age group** |  |  |  |  |  |  |  |
| <65 years | 1952 | 195 (10.0) | 213 (10.9) | 273 (14.0) | 161 (8.3) | 198 (10.1) | 202 (10.4) |
| ≥65 years | 1053 | 125 (11.9) | 135 (12.8) | 196 (18.6) | 103 (9.8) | 133 (12.6) | 160 (15.2) |
| **Tumour type** |  |  |  |  |  |  |  |
| CRC group | 2798 | 311 (11.1) | 337 (12.0) | 448 (16.0) | 257 (9.2) | 320 (11.4) | 347 (12.4) |
| Fruq. Monotherapy | 1888 | 192 (10.2) | 220 (11.7) | 322 (17.1) | 160 (8.5) | 208 (11.0) | 256 (13.6) |
| Fruq. Combination therapy^a^ | 910 | 119 (13.1) | 117 (12.9) | 126 (13.9) | 97 (10.7) | 112 (12.3) | 91 (10.0) |
| with ICI | 493 | 62 (12.6) | 69 (14.0) | 58 (11.8) | 51 (10.3) | 66 (13.4) | 37 (7.5) |
| with Chemo | 359 | 49 (13.7) | 49 (13.7) | 49 (13.7) | 41 (11.4) | 48 (13.4) | 38 (10.6) |
| Non-CRC group | 204 | 9 (4.4) | 11 (5.4) | 21 (10.3) | 7 (3.4) | 11 (5.4) | 15 (7.4) |
| **Metastasis site** |  |  |  |  |  |  |  |
| Lung | 1238 | 168 (13.6) | 177 (14.3) | 198 (16.0) | 129 (10.4) | 174 (14.1) | 152 (12.3) |
| Liver | 1733 | 187 (10.8) | 200 (11.5) | 292 (16.9) | 155 (8.9) | 193 (11.1) | 225 (13.0) |
| Lung and liver | 609 | 89 (14.6) | 86 (14.1) | 108 (17.7) | 66 (10.8) | 86 (14.1) | 80 (13.1) |
| **Baseline ECOG PS score**^b^ |  |  |  |  |  |  |  |
| CRC group |  |  |  |  |  |  |  |
| 0–1 | 138 | 19 (13.8) | 18 (13.0) | 16 (11.6) | 16 (11.6) | 17 (12.3) | 11 (8.0) |
| ≥2 | 33 | 3 (9.1) | 1 (3.0) | 6 (18.2) | 3 (9.1) | 1 (3.0) | 3 (9.1) |
| **Initial dose level of fruquintinib** |  |  |  |  |  |  |  |
| 5 mg | 1923 | 225 (11.7) | 258 (13.4) | 335 (17.4) | 187 (9.7) | 247 (12.8) | 257 (13.4) |
| 4 mg | 303 | 30 (9.9) | 47 (15.5) | 41 (13.5) | 26 (8.6) | 43 (14.2) | 36 (11.9) |
| 3 mg | 730 | 60 (8.2) | 38 (5.2) | 87 (11.9) | 48 (6.6) | 36 (5.0) | 64 (8.8) |

^a^The immunotherapies given in combination with fruquintinib included PD-1 inhibitors sintilimab, camrelizumab, and tislelizumab, and PD-L1 inhibitors durvalumab and atezolizumab. The chemotherapy agents that were given together with fruquintinib included capecitabine, trifluridine/tipiracil, and tegafur/gimeracil/octeracil administered orally, and oxaliplatin, raltitrexed, fluorouracil, and irinotecan administered intravenously.

^b^ECOG PS score were only collected from enrolled patients under protocol version 2.0.
Chemo, chemotherapy; CRC, colorectal cancer, ECOG PS, Eastern Cooperative Oncology Group performance score; Fruq., fruquintinib; ICI, immune checkpoint inhibitor; TEAE, treatment-emergent adverse event; TRAE, treatment-related adverse event.
